# Cross-sectional study assessing the performance of the Arabic translated childhood asthma control test

**DOI:** 10.1038/s41533-018-0109-3

**Published:** 2018-11-01

**Authors:** Majid AlTeneiji, Alia AlKalbani, Huda Nasser, Durdana Iram, Afaf Alblooshi, Hassib Narchi

**Affiliations:** 1 Department of Pediatrics, Tawam Hospital in affiliations with Johns Hopkins Medicine, P.O.Box 15258, Abu Dhabi, United Arab Emirates; 2 Department of Pediatrics, College of Medicine and Health Science, United Arab Emirates University, P.O.Box 17666, Al-Ain, United Arab Emirates

## Abstract

The standard Arabic version of the Childhood Asthma Control Test (C-ACT) has never been previously evaluated in Arab countries. We studied its correlation in Arabic speaking children in the United Arab Emirates (UAE), with both the GINA assessment of asthma control and the resulting changes in asthma management. The Arabic C-ACT was completed by the children or by their parents when needed. A GINA based level of asthma control score was assigned by their managing physician. The correlation between the different cut- scores of the C-ACT and GINA were studied. A total of 105 eligible children with asthma (aged between 4 and 11.8 years, 61% boys) were enrolled. The Arabic translated C-ACT had a high reliability (Cronbach alpha 81%) and validity (as it correlated well with the GINA level of control). We found that using it with the traditional cut-score of 19 overestimated the degree of asthma control. Instead, a calculated optimal cut-score of 20 estimated more accurately the level of asthma control as assessed both by the GINA assessment and also by changes in asthma management. The current Arabic version of the C-ACT has a good reliability and validity. By using a single optimal cut-point of 20, it can be used to assess both the level of asthma control and of treatment control. It does not, however, accurately define asthma control when using the originally proposed cut-score of 19. Physicians need to recognise that the C-ACT cut-points may vary in different populations. We suggest that cut-scores of translated versions need to be modified in different geographical settings.

## Asthma: Improving test accuracy for different populations

The cut-off score suggestive of poor disease management in the Childhood Asthma Control Test (C-ACT) may need to be changed for different geographical populations. Majid AlTeneiji at Tawam Hospital in Al-Ain, and co-workers across the United Arab Emirates compared the performance of the Arabic version of the C-ACT with the global asthma control test, GINA, in verifying levels of asthma and treatment control. The researchers enrolled 105 children aged 4 to 11 who completed both tests. The Arabic C-ACT performed with high reliability and validity, but the team found that the original English cut-point score of 19, indicating poor disease management, should be raised to 20 in their Arabic population to improve test accuracy. AlTeneiji’s team suggest that the C-ACT test points system may need to be revised for different geographical settings around the world.

## Introduction

Asthma is the most common chronic disease in children^1^ with an estimated prevalence of 6 to 13% in those aged between 6 and 14 years in the United Arab Emirates (UAE).^2,3^ It is a leading cause of school absenteeism when poorly controlled.^4^

The assessment of asthma control in children plays an important role in asthma management. It now replaces asthma severity to aid asthma management in the most recent Global Initiative for Asthma (GINA) guideline.^5^ Assessment of asthma control based on symptoms in children is challenging, as it is essentially subjective, depending on the reliability of the parent’s perception of their child’s airway obstruction,^6^ which may differ from the physician’s perception of asthma control, which is also subjective.^7^

Multiple questionnaires have already been introduced into clinical practice to aid the assessment of asthma control in children. One example is the Childhood Asthma Control Test (C-ACT) questionnaire, increasingly implemented in clinical settings for children between the ages of four and eleven years.^8^ It is completed by the parents or by the children themselves. The concordance between C-ACT and the GINA levels of asthma control is not always optimal.^9,10^

The C-ACT has been translated into different languages,^11–14^ including Arabic. The standard classical Arabic language is the official language that is formally taught, read, written and understood across the Arab world. However, as a multitude of only spoken dialects, often with different pronunciations, exist in different Arab countries and even within the same country, the spoken Tunisian dialect for instance is unlikely to be fully understood in other Arab countries and vice-versa. For example, the classical standard Arabic name for tomorrow is “ghadan”, it is pronounced in different dialects throughout the Arab word, such as “bokra” or “bacher”. Therefore, often, an effective verbal conversation between people from different Arab countries necessitates the use of the classical Arabic instead of their respective dialect. This is somewhat similar to the American words “faucet, car hood and trunk” compared to the British exact equivalent in “tap, car bonnet and boot”. Although the Arabic version of the C-ACT has been validated using the Tunisian Arabic dialect,^15^ the performance of that instrument in other Arabic speaking countries is likely to be very different.

As errors in the management of children with asthma might result from incorrect classification of the level of control derived from the C-ACT questionnaire, it is imperative to analyze the performance of the Arabic version of that instrument in a population that has its own Arabic dialect. We therefore analyzed in Arabic speaking children in the UAE, the correlation between the standard Arabic version of the C-ACT and the GINA assessment of asthma control, as well as with the resulting modifications made in the management of asthma.

## Results

One hundred and five children aged between 4 and 11.8 years (mean age 7 years, 61% boys) were enrolled in the study. Their anthropometric data, their C-ACT score, their category of GINA assessments of control and the resulting modifications of their asthma management are shown in Table 1.

**Table 1:** Sample characteristics of the study population (*n* = 105)

Age (y)	
Mean ± SD (median)	7.9 ± 2.4 (7.8)
Range	4.0–11.8
Anthropometrics	
Weight (kg)	28.5 ± 11.7
Height (cm)	126.1 ± 14.5
BMI (kg/m^2^)	17.2 ± 4.2
C-ACT No. (%)	
Controlled	47 (45)
Uncontrolled	58 (55)
Specialist assessment of control using GINA No. (%)	
Well controlled	34 (32)
Partly controlled	23 (22)
Uncontrolled	48 (46)
Specialist assessed change in therapy No. (%)	
Step down	2 (2)
No change	44 (42)
Step up	59 (56)

*BMI* body mass index, *C-ACT* Childhood Asthma Control Test, *GINA* Global Initiative for Asthma

**Fig. 1:**
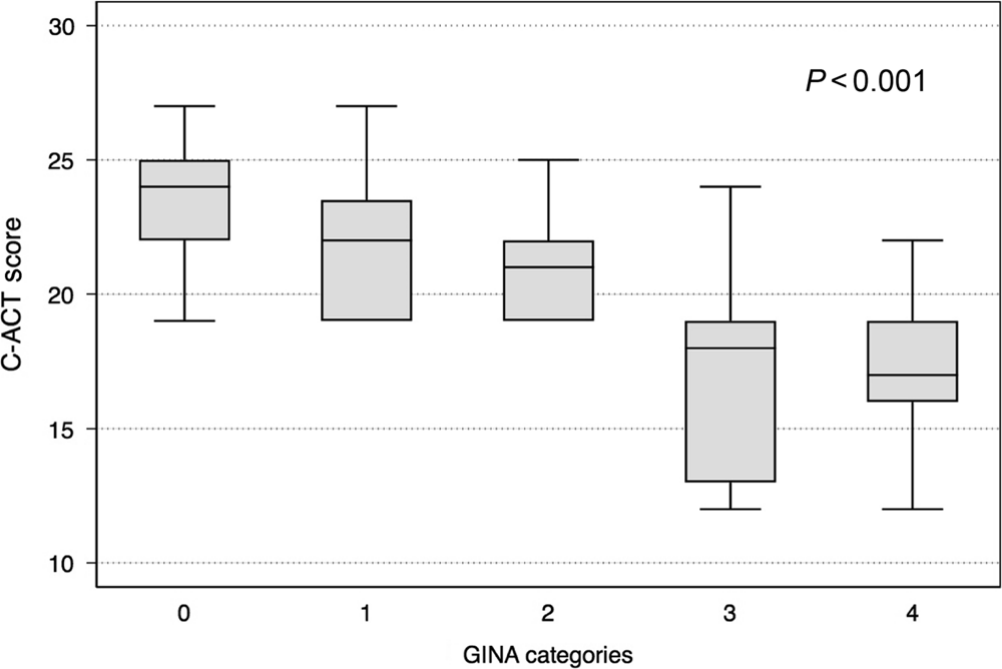
The C-ACT score correlation with the GINA scoring

**Fig. 2:**
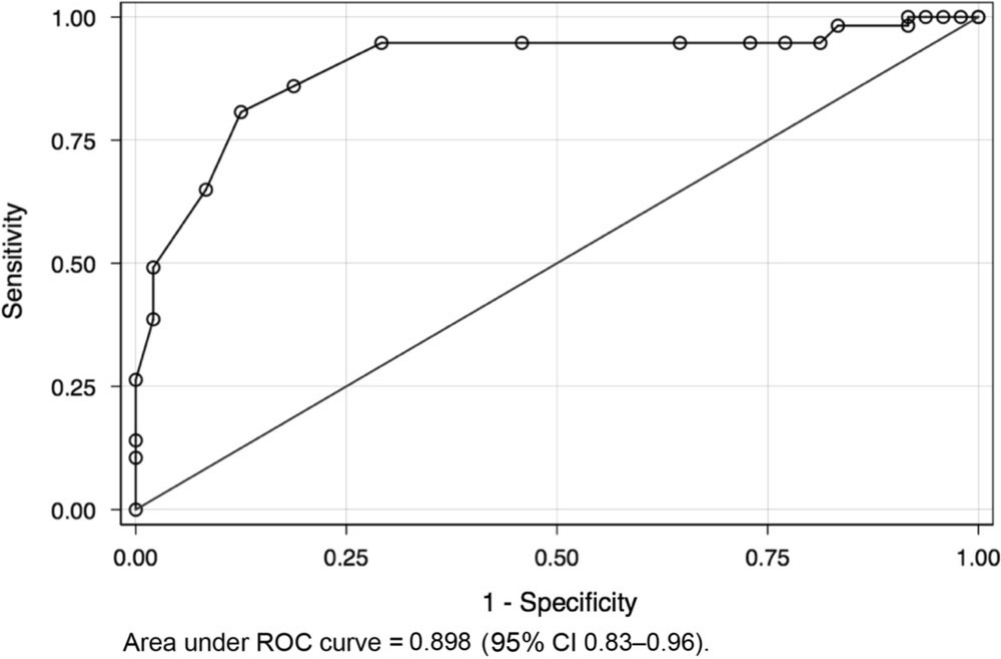
ROC curve of C-ACT scores by GINA asthma control categories (well controlled versus all other categories)

The Cronbach alpha estimate for the internal consistency or reliability of seven items Arabic C-ACT survey was 0.81% in the total sample, indicating high consistency and reliability of that tool. The C-ACT score correlated well (*P* < 0.001) with the GINA scoring confirming also its validity (Fig. 1 and Table 2S). Using a C-ACT cut-score of 19 to classify the level of control by GINA had a sensitivity of 94.74%, a specificity of 70.83%, a Youden index of 0.65 and correctly classified GINA control in nearly 84% of the children (Table 2). A C-ACT score of 21 showed the highest Youden index (0.68) with a sensitivity of 80.70%, a specificity of 87.50% and similarly correctly classified GINA control in nearly 84% of the children (Table 2). The respective receiver operating characteristic (ROC) curve (Fig. 2) yielded an area under curve (AUC) of 0.898 (95% CI 0.83–0.96). Using the Youden method the optimal cut-point to classify GINA control categories was a C-ACT score of 20 (standard error 0.8, 95% CI 18.3–21.6, *P* < 0.001).

**Table 2:** Receiver operator characteristic (ROC) curve of C-ACT score performance in classifying GINA categories (well controlled v/s all other categories) in 105 children with asthma

Cut-point value of C-ACT score	Sensitivity %	Specificity %	Youden Index	Correctly classified %	Positive likelihood ratio	Negative likelihood ratio
9	100.00	6.25	0.0625	57.14	1.0667	0
11	100.00	8.33	0.0833	58.10	1.0909	0
12	98.25	8.33	0.0658	57.14	1.0718	0.2105
13	98.25	16.67	0.1492	60.95	1.1789	0.1053
14	94.74	18.75	0.1349	60.00	1.166	0.2807
15	94.74	22.92	0.1766	61.90	1.229	0.2297
16	94.74	27.08	0.2182	63.81	1.2992	0.1943
17	94.74	35.42	0.3016	67.62	1.4669	0.1486
18	94.74	54.17	0.4891	76.19	2.067	0.0972
19	94.74	70.83	0.6557	83.81	3.2481	0.0743
20	85.96	81.25	0.6721	83.81	4.5848	0.1727
21	80.70	87.50	0.682	83.81	6.4561	0.2206
22	64.91	91.67	0.5658	77.14	7.7895	0.3828
23	49.12	97.92	0.4704	71.43	23.579	0.5196
24	38.60	97.92	0.3652	65.71	18.5264	0.6271
25	26.32	100.00	0.2632	60.00		0.7368
26	14.04	100.00	0.1404	53.33		0.8596
27	10.53	100.00	0.1053	51.43		0.8947

Area under ROC curve = 0.89, SE = 0.03, 95 confidence intervals 0.83–0.96

*C-ACT* Childhood Asthma Control Test, *GINA* Global Initiative for Asthma

**Fig. 3:**
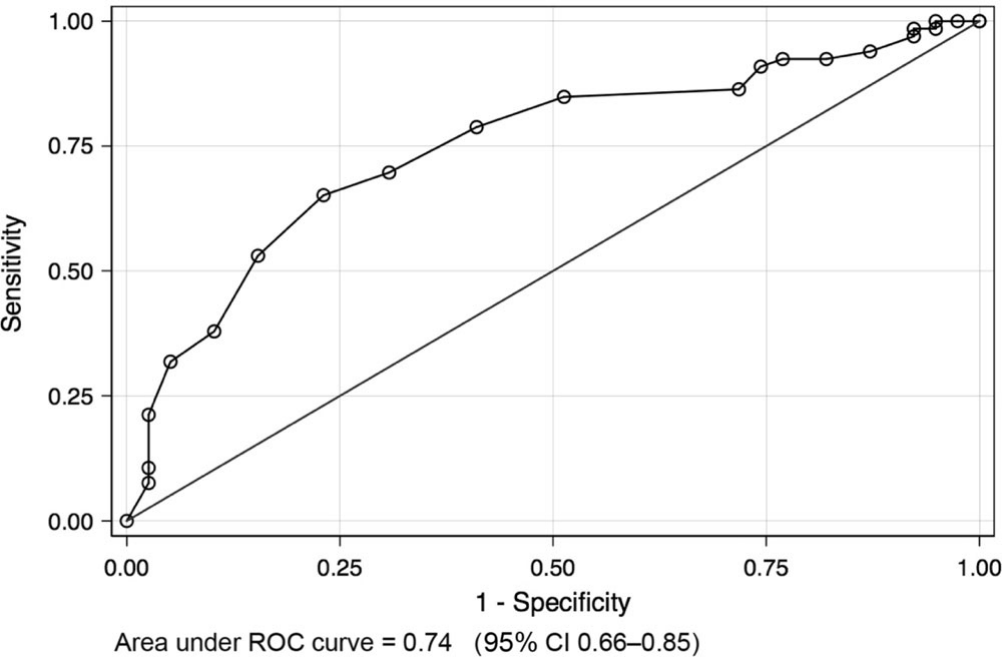
ROC curve of C-ACT scores by treatment change categories (well controlled versus all other categories)

The C-ACT also correlated well with the resulting change in therapy in 73 participants 70% (Table 3S). A C-ACT cut-score of 19 had a sensitivity of 78.79%, a specificity of 58.97%, a Youden index of 0.3776 and correctly classified the treatment change category in 71% of children, while a score of 21 has a sensitivity of 65.15%, a specificity of 76.92%, a Youden index of 0.42 and correctly classified over 69% of the children (Table 3). The AUC of the respective ROC curve was 0.749 (95% CI 0.66–0.85) (Fig. 3). The optimal cut-point using the Youden method to classify treatment change categories was a score of 20 (standard error 1.2, 95% CI 17.5–22.4, *P* < 0.001).

**Table 3:** Receiver operator characteristic (ROC) curve of C-ACT score performance in classifying treatment change categories (well controlled v/s all other categories) in 105 children with asthma

Cut-point value of C-ACT score	Sensitivity %	Specificity %	Youden index	Correctly classified %	Positive likelihood ratio	Negative likelihood ratio
9	98.48	5.13	0.0361	63.81	1.0381	0.2955
11	98.48	7.69	0.0617	64.76	1.0669	0.197
12	96.97	7.69	0.0466	63.81	1.0505	0.3939
13	93.94	12.82	0.0676	63.81	1.0775	0.4727
14	92.42	17.95	0.1037	64.76	1.1264	0.4221
15	92.42	23.08	0.155	66.67	1.2015	0.3283
16	90.91	25.64	0.1655	66.67	1.2226	0.3545
17	86.36	28.21	0.1457	64.76	1.2029	0.4835
18	84.85	48.72	0.3357	71.43	1.6545	0.311
19	78.79	58.97	0.3776	71.43	1.9205	0.3597
20	69.70	69.23	0.3893	69.52	2.2652	0.4377
21	65.15	76.92	0.4207	69.52	2.8232	0.453
22	53.03	84.62	0.3765	64.76	3.447	0.5551
23	37.88	89.74	0.2762	57.14	3.6932	0.6922
24	31.82	94.87	0.2669	55.24	6.2045	0.7187
25	21.21	97.44	0.1865	49.52	8.2727	0.8086
26	10.61	97.44	0.0805	42.86	4.1364	0.9175
27	7.58	97.44	0.0502	40.95	2.9545	0.9486

Area under ROC curve = 0.75, SE = 0.05, 95 confidence intervals 0.65–0.84

*C-ACT* Childhood Asthma Control Test

The cut-point scores for classifying both the GINA and the treatment control (as well as their respective 95% confidence intervals) were quasi similar and showed no statistically significant difference (*P* = 1.0), highlighting that a single Arabic C-ACT cut-point of 20 can be used to adequately classify both levels of control.

## Discussion

Comparing the standard Arabic version of the C-ACT to the GINA assessment of asthma control, we found it to have high reliability as well as validity, as it correlated well with the GINA criteria of asthma control. However, using the recommended cut-point of 19 limit overestimated asthma control in our population. This confirms previous reports where other versions of the C-ACT were used.^10,14,16,17^

We found that a C-ACT score of 20 was optimal to accurately estimate the level of asthma control rather than the recommended traditional cut off limit of 19 in our population. This also validates previous reports which had used the Spanish and French versions^14,16^ as well as a study from Tunisia which used the Tunisian Arabic dialect version of the C-ACT.^15^ As the majority of previous publications have only reported the Youden index but without its 95% CI, it is not possible to statistically compare their optimal score with ours. Other studies, using different languages to English and comparing C-ACT to physician assessment, asthma diaries and/or objectives measures such as spirometry or fractional exhaled nitric oxide, also found that the most appropriate C-ACT cut-offs scores ranged from 20 up to 24, but unfortunately without providing confidence intervals.^10,11,14,16,17,18–22^ The differences in the cut-scores between GINA and C-ACT are a direct reflection that they are two different tools: while GINA categorizes control based on frequency of symptoms, the C-ACT focuses on their severity. The differences among all the suggested C-ACT cut-off scores by the different studies^19–21^ is probably underpinned by differences in the perception and understanding of asthma control, resulting most likely from differences in cultural and educational backgrounds between the various studied populations. This finding suggests that the proposed cut-off value to define asthma control in the English version of the C-ACT, is different from those where other languages are used.

Although we found a significant correlation between the C-ACT and change in asthma treatment at a cut-point of 19, the Youden index was the highest at a cut-point of 21. However, the computed optimal cut-point was 20, making it therefore more appropriate in our population than the recommended cut-point of 19, as confirmed in earlier studies.^16,18,19,22^

Interestingly, the cut-point scores for classifying both the GINA and the treatment control were identical, highlighting that a single Arabic C-ACT cut-point of 20 can be used to adequately classify both levels of control, simplifying considerably its use.

One of the limitations in our study was the small sample size. Another was the lack of objective measures to assess asthma control, such as spirometry, although that limitation is probably mitigated by conflicting results of correlation studies between spirometry and asthma control.^11,12,23,24^ Because of the difficultly in using spirometry in young children, there is a need for future studies which will use other objective measures not requiring the child’s cooperation such as the forced oscillation technique (FOT). Furthermore, we believe a longitudinal assessment to apply a test–retest analysis will add more strength to future studies. As the majority of previous studies have not calculated or reported their optimal cut-point with 95% confidence intervals, a formal statistical comparison of the optimal cut-points amongst all the studies was not possible. We encourage future studies to use the methodology which we have used to enable robust comparison of the recommended optimal scores.

The Arabic translated C-ACT had a high reliability and validity. However, the recommended cut-points of the original English version do not accurately define asthma control in native children in the UAE. A single Arabic C-ACT cut-point of 20 correctly classifies the level of control both by GINA or by treatment control, simplifying considerably its use. We believe that, in different populations, especially with different languages, the optimal C-ACT score cut-point should be specifically calculated to accurately establish asthma control in native children.

## Methods

### Participants

Emirati national children, aged between 4 and 11 years, having Arabic as their native language, were eligible for enrolment in the study if they had a physician diagnosis of asthma. All study participants were managed by pediatric pulmonology staff in the outpatient pediatric pulmonology clinic at Tawam hospital (Al-Ain city, UAE) between April 2015 until April 2017 and were already on prophylactic therapy for asthma at the discretion of their treating physician.

All participating children had their height and weight measured, and they (or their parents, when appropriate) were requested to complete the Arabic C-ACT questionnaire. The children were then seen by a pediatric pulmonologist who assessed the level of asthma control using the GINA guidelines.^5^ All the described data were obtained in a single visit.

Exclusion criteria includes any systemic illness, seizure disorder, congenital anomaly, cerebral palsy, chest surgery, chronic lung disease of infancy, and upper airway abnormality.

### Sample size

According to our audit results, an estimated 5–10% of children with asthma (average 7.5%) attending our clinic are not well controlled. We have therefore calculated that a minimum sample size of 101 participants is required to give the study enough power to detect a 5% difference in disease control with a precision of 5 and 95% confidence level. We decided to enroll 105 children to compensate for eventual attrition.

### Instrument: Arabic childhood asthma control test

C-ACT is composed of seven questions (four child-reported and three parent-reported) with a score range from zero (poor control) to 27 (complete control). A score ≤19 defines uncontrolled asthma and, when ≤12 the condition is defined as very poorly controlled.^8,13^ The Heath Authority of Abu Dhabi, which governs all government hospitals in the emirate, translated the C-ACT into Arabic, approved the translated tool and recommended its use in all its affiliated health institutions, including ours, in 2014.

### GINA assessment of asthma control

In GINA assessment of asthma control the physician evaluates asthma control by assessing the presence of the following symptoms in the 4 preceding weeks: (1) presence of daytime asthma symptoms, (2) nighttime asthma symptoms, (3) activity limitation and (4) use of short acting β2-agonist. The resulting level of asthma control is classified as (1) well controlled if the child does not have any of the above symptoms, (2) partially controlled in the presence of one or two of the above symptoms and (3) uncontrolled in the presence of at least three of the above symptoms.^5^

### Change in therapy based on physician assessment of asthma control

Using their respective physician’s discretion to modify the therapeutic plan, according to the physician assessment, the participants were categorized into three groups: (1) step down (reducing inhaled steroid dose/long acting beta agonists (LABA) or Montelukast), (2) no modification to the management, and (3) step up in therapy, consisting of either (a) increasing the dose of inhaled steroid dose or the addition of LABA or Montelukast if the children were deemed to be uncontrolled, or (b) maintaining the same medication if the participants were deemed to be non-compliant or if their medication administration technique was suboptimal. For the analysis, the number of categories was reduced to only two: (1) controlled group (if medications were stepped down or not changed) and (2) uncontrolled group (if medications were stepped up).

### Ethics approval

The study was approved by the institutional research review board (ref: CRD 531/17 -AAMDHREC Protocol No. 531–17). All research was conducted in accordance with all relevant guidelines and procedures.

### Statistical analysis

The reliability, or internal consistency of the seven items on C-ACT was assessed by the Cronbach’s alpha test. Its validity was evaluated by the degree of correlation it had with the score of the GINA assessment of asthma control.

Normally distributed continuous variables were reported as mean ± standard deviation (SD) and categories distribution as number of participants (and percentage).

The univariate analysis of continuous variables (C-ACT and GINA) between the categories was conducted with the Analysis of Variance test (ANOVA). Proportions were compared with the *χ*^2^ test and the Fisher exact test was used for small proportions.

The sensitivity, specificity, Youden index, percentage of correct classification, positive and negative likelihood ratios were calculated for the different cut-scores of the C-ACT to categorize the GINA assessment as well as the categorization of the change in asthma management. The resulting receiver operator characteristic (ROC) curves were constructed and their respective area under the ROC curve (AUC), with 95% confidence intervals (CI), were reported. In addition, using the Youden method, the optimal cut-score (with 95% CI) was computed using bootstrapping with 100 replications.

All the analyses were performed with the STATA statistical package version 14 (StataCorp, College Station, TX, USA) and statistical significance was defined by a two-sided *p*-value < 0.05.

## Electronic supplementary material

Supplementary Material

## Data Availability

All data generated or analyzed during this study are included in this published article (and its supplementary information files).

## Electronic supplementary material

**Supplementary information** accompanies the paper on the *npj Primary Care Respiratory Medicine* website (10.1038/s41533-018-0109-3).
