# Supplementary material for: Cross-sectional study assessing the performance of the Arabic translated childhood asthma control test
Source: NPJ Prim Care Respir Med. 2018 Nov 1;28:41. doi: 10.1038/s41533-018-0109-3 (PMC6212419; doi:10.1038/s41533-018-0109-3)
Supplement: Supplementary file 1 — Supplementary Material [file 41533_2018_109_MOESM1_ESM.pdf]

Table 1S. Summary of the C-ACT and GINA score in the study cohort (n=105).

C-ACT questions score	0	1	2	3	4	5
Q1- How is your asthma today?	1 (0.9)	15 (14.3)	49(46.7)	40 (38.1)	-	-
Q2- Problem when you run, exercise or play sports?	6 5.7)	14 (13.3)	57 (54.3)	28 (26.7)	-	-
Q3- Cough because of asthma?	13 (12.4)	30 (28.6)	49 (46.7)	13 (12.4)	-	-
Q4- Wake up during the night because of your asthma?	7 (6.7)	25 (23.8)	46 (43.8)	27 (25.7)	-	-
Q5- Number of days with daytime asthma symptoms over 4 weeks?	4 (3.8)	3 (2.8)	5 (4.7)	19 (18.1)	39 (37.1)	35 (33.3)
Q6- Number of days over 4 weeks with wheeze during the day because of asthma	3 (2.8)	4 (3.8)	-	16 (15.2)	34 (32.4)	48 (45.7)
Q7- Number of days over 4 weeks waking up during the night	5 (4.7)	3 (2.8)	-	14 (13.3)	37 (35.2)	46 (43.8)
C-ACT mean total score ± SD			19.6 ± 4.9			

GINA questions score	0	1
Daytime Symptoms	50 (47.6)	55 (52.4)
Limitation of activity	50 (47.6)	55 (52.4)
Nocturnal symptoms	54 (51.4)	51 (48.6)
Need for rescue treatment	51 (48.6)	54 (51.4)

Data are presented as a number (percent) unless stated otherwise
C-ACT (Childhood Asthma Control Test), GINA (Global Initiative for Asthma)

Table 2S. Mean (SD) C-ACT score by GINA score categories in 105 children with asthma

GINA score	Number of children	Mean SD C-ACT score
0	34	23.55 (2.24)
1	12	21.25 (4.07)
2	11	19.81 (3.71)
3	11	17.00 (4.00)
4	37	16.24 (4.65)

Analysis of variance ($P < 0.001$)
C-ACT (Childhood Asthma Control Test)
GINA (Global Initiative for Asthma)

Table 3S. Distribution of C-ACT and management control categories in 105 children with asthma, expressed as number

	C-ACT		
Management	Controlled	Uncontrolled	Total
Uncontrolled	12	27	39
Controlled	46	20	66
Total	58	47	105

P<0.001 (Chi^2 test)
C-ACT (Childhood Asthma Control Test)
